# Erectile function after different techniques of bulbar urethroplasty: does urethral transection make a difference?

**DOI:** 10.1186/s12894-023-01281-y

**Published:** 2023-08-24

**Authors:** Osama Shalkamy, Mohamed Elsalhy, Saleh Mohammed Alghamdi, Mohammed Beaiti, Ibrahim Abdel-Al, Mahmoud Faisal, Tamer A. Abouelgreed, Yasser Badran, Abdrabu Abdrabu, Mahdi Al-Ayafi, Mohanad Jebril Bosily, Salah E. Shebl, Ibrahim nematallah, Ahmed Shafiea, Adel Elatreisy

**Affiliations:** 1 Department of Urology, Faculty of Medicine, Al-Azhar University, Cairo, Egypt; 2 Department of Urology, Armed Forces Hospital Southern Region, Khamis Mushait, Saudi Arabia; 3 Department of Urology, Faculty of Medicine, Assiut Branch, Al-Azhar University, Assiut, Egypt

**Keywords:** Stricture urethra, Urethroplasty, Erectile dysfunction, Postoperative complications

## Abstract

**Purpose:**

We aimed to compare the impact of urethral transection after different techniques of bulbar urethroplasty on erectile function outcome.

**Materials and methods:**

We retrospectively reviewed the records for 245 patients who underwent different urethroplasty techniques for bulbar urethral stricture between February 2013 and January 2021. The comparison between the transecting and non-transecting cohorts included patients’ demographics, clinicopathological features of the urethral stricture, post-urethroplasty erectile function, and success of urethroplasty. Outcomes were erectile function status verified by IIEF5-15 score at preoperative, three months, and 12 months post-surgery. We defined Post-urethroplasty ED as a decrease of 5 points or more.

**Results:**

The urethroplasty success rate of the entire cohort was 86.9% after a mean follow-up of 45.59 ± 21 months. Out of 245 patients, 18 (7.3%) experienced 90-day complications. Transecting bulbar urethroplasty techniques were performed in 74 patients (30.2%), while non-transecting techniques were performed in 171 patients (69.8%). there were no differences between the cohorts regarding urethroplasty success (87.8% Vs. 86.5%, Mantel-Cox test p = 0.93) or postoperative complications (8.1% Vs. 7%, p = 0.73). Transient ED was evident in the transecting cohort as reported in 8.1% compared to 2.9% for the non-transecting (p = 0.07).Still, but de novo permanent ED was comparable (4.1% Vs. 2.9%, p = 0.65), for transecting and non-transecting, respectively.

**Conclusions:**

Unfortunately, some patients who undergo transecting techniques of bulbar urethroplasty experience transient erectile dysfunction that can improve within the first post- urethroplasty year; however, de novo permanent erectile dysfunction is uncommon after different techniques of bulbar urethroplasty and is not predisposed by urethral transection.

## Introduction

The best bulbar urethroplasty technique is still controversial. Many surgeons prefer non-transecting urethroplasty techniques based on the proximity of the bulbar urethra to the neurovascular penile bundles; such urethral transection carries the risk of post- urethroplasty ED (erectile dysfunction) [1]. In BMG (buccal mucosal graft) Bulbar urethroplasty, transient ED could rely on transient neurapraxia, and blood supply alteration that revascularizes in the healing process. However, permanent ED could be explained by cavernous and perineal nerve injury and bulbar artery flow disruption [2]. Al- Qudah and Santucci reported the superiority of BMG bulbar urethroplasty over the anastomotic repair with a better functional outcome and no reported sexual dysfunction compared to 18% after anastomotic repair [3]; also, many studies reported a variety of erectile and ejaculatory dysfunctions following transecting anastomotic urethroplasty [1, 4]. Conversely, many other surgeons consider EPA (excision and primary anastomosis) with the corpus spongiosum transection and scared tissue excision as the preferred technique for short bulbar stricture urethra as it offers a durable outcome with low morbidity [5, 6], in addition to many reports stating that erectile dysfunction following transecting urethroplasty is uncommon [7–11]. Controversy in urethral transection, besides the preference for anastomotic urethroplasty by many surgeons, resulted in the development of a non- transecting anastomotic urethroplasty approach that abandons the risk of penile neurovascular damage while keeping the efficient end to end anastomosis [12, 13]. The present study compared the transecting and non-transecting bulbar urethroplasty techniques regarding erectile function and urethral patency outcomes.

## Patients and methods

A non-concurrent cohort study included all patients who underwent different bulbar urethroplasty techniques at a tertiary care institute in Egypt from February 2013 to January 2021, after the study protocol approval from the institutional review board. We excluded sexually inactive patients with preoperative moderate or severe ED (IIEF5-15 score < 19) [14], older than sixty-five, and younger than eighteen. The study population was subdivided into two groups. Group I included patients who underwent transecting bulbar urethroplasty techniques, including EPA (n = 36), augmented anastomotic (n = 14), dorsal BMG with ventral penile skin flap (n = 6), and staged urethroplasty (n = 18). Group II included patients who underwent non-transecting bulbar urethroplasty techniques, including dorsal onlay BMG (n = 103), ventral onlay BMG (n = 62), dorsal inlay BMG (n = 3), double face BMG (n = 2), and Non-transecting anastomotic bulbar urethroplasty (n = 1). All surgical procedures were done by two teams supervised by an expert urethral surgeon. The intraoperative choice for different urethroplasty techniques was based predominantly on the urethral stricture length, the status of the urethral plate, the degree of spongiofibrosis, and previous urethral procedures. The ventral onlay BMG technique was done as described by Wessells H [15], and dorsal onlay BMG as described by Barbagli et al. [16]. BMG was harvested as described by Morey and McAninch [17]. Transecting EPA included complete transection of the corpus spongiosum with stricture excision and a standard spatulated tension-free anastomosis.

## Follow up and outcome

Patients were booked for clinic visits after three weeks from surgery for clinical evaluation and catheter removal proceeded by peri-catheter urethrogram. All patients were booked for prospective follow-up visits, including clinic visits and uroflowmetry at three-month intervals in the first-year post-surgery, then annually, urethrogram after six months, and 17 French semirigid cystoscopy after nine months. Patients who experienced voiding LUTS or had low Q max ≤ 14 mL/s were evaluated immediately with urethrogram ± cystoscopy. Urethroplasty success was defined as the absence of voiding lower urinary tract symptoms and confirmation of continued urethral patency with no need for further intervention, including urethral dilatation. The comparison between the cohorts was made according to the data retrieved, including patients’ demographic and the clinicopathological features of the urethral stricture. We used two measures to evaluate the voiding function: the International Prostate Symptom Score (IPSS) and maximum urinary flow (Qmax). Additionally, we evaluated preoperative erectile function using the International Index of Erectile Function questionnaire (IIEF5-15) (Q1,2,3,4,5,15) [18]. Intraoperative recording of operative time and harvested BMG length, the stricture length was identified by intra-operative measurements; moreover, perioperative complications and postoperative outcomes, including urethral stricture recurrence and post urethroplasty erectile function status, verified by IIEF5-15 questionnaire after 3 and 12 months. We defined Post-urethroplasty ED as a decrease of 5 points or more on the IIEF5-15 questionnaire compared to the pre-urethroplasty score.

### Statistical analysis

The Cox regression analysis and Mantel-Cox test compared transecting and non-transecting techniques regarding time to failure. Data analysis was done by utilizing the Statistical Package for Social Science (SPSS) software, version 27 (SPSS Inc., Chicago, IL, USA). Categorical variables were presented as frequency and percentage, whereas numeric variables were presented as a mean and standard deviation. The Chi-square test was used to test the association between two categorical variables. At the same time, the student’s t-test was applied to test for the difference in the means of continuous variables between two different groups. The paired-samples t-test was used to detect the significance level between preoperative and follow-up data in the same group. The P < 0.05 was considered significant.

## Results

**Fig. 1:**
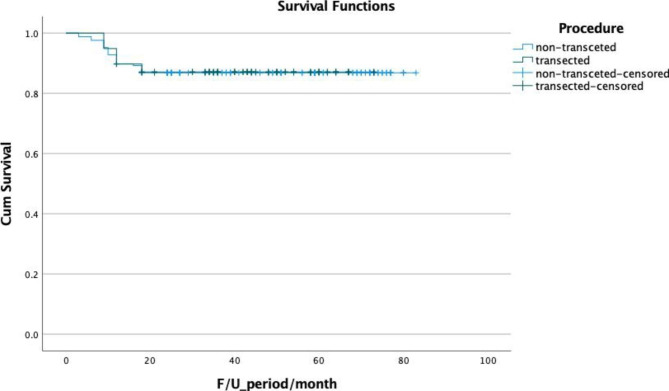
Mantel-Cox analysis of urethroplasty success between transecting and non-transecting techniques

Two hundred forty-five patients met our inclusion criteria with complete follow-up data. The mean urethral stricture length was 3.7 cm. The most common cause for urethral stricture was trauma in 32.3% (n = 79) followed by iatrogenic causes in 30.2% (n = 74), idiopathic in 25.3% (n = 62) and inflammatory in 12.2% (n = 30). Out of 245 patients, 18 (7.3%) experienced 90-day complications with a Clavien grade more than one, including post-urethroplasty wound infection in 13 patients (5.3%), perineal hematoma in 3 cases (1.2%), and orchitis in 2 cases (0.8%). The urethroplasty success rate of the entire cohort was 86.9% after a mean follow-up of 45.59 ± 21 months. Transecting bulbar urethroplasty techniques were performed in 74 patients (30.2%), and non-transecting techniques were performed in 171 patients (69.8%), as listed in Table 1. The cohorts were comparable regarding patients’ demographics and urethral stricture criteria apart from a statistically significant longer stricture segment in the non-transecting cohort and lower Q-max in the transecting cohort, as illustrated in Table 2. The mean operative time, hospital stay, and catheter duration were significantly longer in the non-transecting cohort, as shown in Table 3. Mantel-Cox analysis showed no statistically significant difference regarding urethroplasty success between transecting and non-transecting techniques (87.8% after a mean follow-up period of 44.29 ± 17.26 Vs. 86.5% after a mean follow-up of 47.6 ± 22.2 months, p = 0.93) (Fig. 1). There was no difference in the preoperative erectile function between transecting and non-transecting cohorts (Table 3); the mean IIEF score significantly decreased after three months from transecting urethral surgery; it dropped from 26.12 to 24.5 (p < 0.001) and improved significantly after one year to 26.24. The mean IIEF score insignificantly decreased after three months from the non- transecting urethral surgery; it dropped from 26.28 to 26.11 (p = 0.16) and insignificantly improved after one year to 26.29 (p = 0.3) (Table 4). In comparison between the cohorts, the mean IIEF score was significantly lower in the transecting after three months from urethroplasty (p < 0.001). Still, it was comparable after one year with an insignificant p-value (Table 3). Transient ED **(**improved within the postoperative year) was significantly higher in transecting cohort as reported in 8.1% of the patients (n = 6/74) compared to 2.9% (n = 5/171) in the non-transecting (p = 0.07). De novo permanent ED was reported in 3.3% of the entire cohort; it was comparable between both techniques as reported in 4.1% and 2.9% for transecting and non-transecting, respectively (Table 3).

**Table 1:** Bulbar urethroplasty techniques used in transected group (I), and non-Transected group (II)

The group	The procedure (n = 245)	Number of Cases	Urethroplasty success, n (%)
Group I (n = 74 cases)	EPA	36 (14.69%)	32 (88.9%)
	Augmented anastomotic urethroplasty	14 (5.71%)	12 (85.7%)
	Dorsal BMG and ventral penile skin flap urethroplasty	6 (2.45%)	4 (66.7%)
	Staged urethroplasty	18 (7.35%)	16 (88.9%)
Group II (n = 171)	Dorsal onlay BMG urethroplasty	103 (42.04%)	91 (88.3%)
	Ventral onlay BMG urethroplasty	62 (25.31%)	54 (87.1%)
	Double face BMG urethroplasty	2 (0.82%)	1 (50%)
	Dorsal inlay BMG urethroplasty (Asopa)	3 (1.23%)	2 (66.7%)
	Non-transecting anastomotic bulbar urethroplasty	1 (0.4)	1 (100%)

**Table 2:** Comparison between Patients’ demographic and the clinicopathological features of the urethral stricture among the study groups

	Group I	Group II	P-value
Age (years)	38.4 ± 15	39.3 ± 13.1	0.65
BMI	23.1 ± 3.9	23.6 ± 3.5	0.32
Smoking, n (%)	21 (26.9%)	37 (22.2%)	0.41
DM, n (%)	10 (12.8%)	16 (9.6%)	0.44
HTN, n (%)	6 (7.7%)	15 (8.9%)	0.74
Previous urethroplasty, n (%)	14 (17.9%)	16 (9.6%)	0.63
Urethral stricture Etiology, n (%)			0.02
Traumatic Iatrogenic Idiopathic Inflammatory	37 (50%) 15 (20.3%) 17 (22.9%) 5 (6.8%)	42 (24.6%) 59 (34.5%) 45 (26.3%) 25 (14.6%)	
Preoperative IPSS	27.5 ± 6.4	25.8 ± 4.8	0.02
Preoperative Q-max	2.5 ± 2.4	4.4 ± 2.8	< 0.001
Stricture length (cm)	3.4 ± 1.8	4.7 ± 1.4	< 0.001

**Table 3:** Comparison between the cohorts regarding intraoperative, postoperative parameters, and erectile function status

	Group I	Group II	p-value
Operative time (min)	140.3 ± 36.3	151.9 ± 4.7	< 0.001
Hospital stays (days)	3.24 ± 0.75	3.55 ± 0.78	0.004
Catheter duration (weeks)	2.9 ± 0.98	3.23 ± 0.4	< 0.001
Post-operative complications			0.69
Wound infection, n (%) Perineal hematoma, n (%) orchitis, n (%)	5 (6.8%) 1 (1.3%) 0	8 (4.7%) 2 (1.2%) 2 (1.2%)	
Post-operative IPSS	4.97 ± 5.59	4.28 ± 4.32	0.29
Post-operative Q-max	21.88 ± 5.8	22.4 ± 6.3	0.54
Follow up period (months)	44.29 ± 17.26	47.6 ± 22.2	0.25
Urethroplasty success, n (%)	65 (87.8%)	148 (86.5%)	0.93
Pre-operative IIEF	26.12 ± 1.7	26.28 ± 1.87	0.4
Preoperative ED, n (%)	11 (14.9%)	20 (11.7%)	0.38
IIEF,3-months postoperative	24.5 ± 2.8	26.11 ± 2.68	< 0.001
Transient ED, n (%)	6 (8.1%)	5 (2.9%)	0.07
IIEF,12-months postoperative	26.24 ± 2.5	26.29 ± 2.47	o.8
Permanent ED, n (%)	3 (4.1%)	5 (2.9%)	0.65

**Table 4:** Postoperative changes in IIEF score in the same study group

Group I	Pre-operative IIEF	3-months postoperative IIEF	p-value
	*26.12 ± 1.7*	*24.5 ± 2.8*	*< 0.001*
	*Pre-operative IIEF*	*12-months postoperative IIEF*	*p-value*
	*26.12 ± 1.7*	*26.24 ± 2.5*	*0.57*
	*3-months postoperative IIEF*	*12-months postoperative IIEF*	*p-value*
	*24.5 ± 2.8*	*26.24 ± 2.5*	*< 0.001*
Group II	*Pre-operative IIEF*	*3-months postoperative IIEF*	*p-value*
	*26.28 ± 1.87*	*26.11 ± 2.68*	*0.16*
	*Pre-operative IIEF*	*12-months postoperative IIEF*	*p-value*
	*26.28 ± 1.87*	*26.29 ± 2.47*	*0.91*
	*3-months postoperative IIEF*	*12-months postoperative IIEF*	*p-value*
	*26.11 ± 2.68*	*26.29 ± 2.47*	*0.3*

The 90-day complication rate did not differ significantly between the cohorts as reported in 6/74 patients (8.1%) in the transecting, opposite to 12/171 patients (7%) in the non-transecting as illustrated in Table 3.

## Discussion

Urethroplasty is widely considered the standard treatment for bulbar urethral stricture and is preferred over endoscopic treatment. It offers a definite, cost-effective, and durable outcome with success exceeding 90% besides low morbidity [1, 8, 19]. Many surgeons have shifted to non-transecting bulbar urethroplasty techniques considering erectile adverse events post- transecting approaches, especially since they have an equal urethral patency success rate [1, 13, 20, 21]. We reported a lower preoperative Q-max in the transecting urethroplasty techniques that could be explained with stricture complexity and severity in post-trauma patients as predominated in the transecting group.

The current study establishes a similar urethroplasty success rate between non- transecting and transecting bulbar urethroplasty procedures exceeding 85% with a comparable low post- urethroplasty morbidity.

In 1993, Mundy first reported erectile dysfunction in 2.5% (5/200) of patients who underwent bulbar or membranous anastomotic urethroplasty that, explained by compromised spongiosal vascular supply and nerves disruption [22], subsequently post-urethroplasty erectile dysfunction was reported in a wide range of patients between 0 and 40% [1–4, 10, 11, 23–28]. Coursey et al. [29] were one of the first authors to evaluate post anterior urethroplasty erectile function utilizing a validated sexual health questionnaire in a retrospective study that included 174 patients who underwent urethroplasty versus circumcision; they found a deterioration including erectile and ejaculatory dysfunction in 31% of patients that improved with time in 61.8%; erectile adverse effects were interestingly similar in the circumcision cohort, it suggests that erectile dysfunction post-urethroplasty is multifactorial. A recent meta-analysis of 36 studies and 2323 patients reported post anterior urethroplasty persistent de novo erectile dysfunction in 1% of cases,

approximately 86% of patients who experience post-anterior urethroplasty ED will improve and return to preoperative sexual status within the postoperative year [28]. Although Many authors suggested urethral transection as a risk factor for post-urethroplasty sexual morbidity [3, 4], still inconsistent in the literature that can be explained by different surgical skills and study populations, variable stricture pathologies, multiple urethroplasty techniques, and even variable postoperative follow-up protocols [22, 24, 30].

Our study reported a significant transient erectile dysfunction following transecting bulbar urethroplasty techniques that improved within the first post-surgery year; it can be explained by postoperative tissue edema that needs time to resolve. The occurrence of this dysfunction is expected to be more in transecting urethroplasty techniques as compared to other non-transecting. This is because it requires more urethral dissection and mobilization, which adds to the risk of nerve damage and blood supply alteration. However, this alteration is compensated with revascularization over time.

The current study approved that permanent de novo erectile dysfunction following bulbar urethroplasty is uncommon (3.3%) and was comparable between transecting and non- transecting techniques. It can have a significant clinical impact as the urethroplasty procedure decision could rely on surgeon preference and urethral stricture features without worries about post- urethroplasty erectile dysfunction.

## The study Limitations

Although our study included a large population of patients who underwent different techniques of bulbar urethroplasty, there are some limitations. First, the study is not prospective or randomized. The cohort differed by etiology as remote trauma predominated in the transecting cohort and needed an excision of scarred tissue—conversely, iatrogenic causes ruled in the non-transecting cohort. Also, the study has unequal distribution among study groups, with a significantly longer stricture segment in the non-transecting urethroplasty techniques and a lower Q-max in the transecting techniques.

## Conclusions

Unfortunately, some patients who undergo transecting techniques of bulbar urethroplasty experience transient erectile dysfunction that can improve within the first post-urethroplasty year; however, de novo permanent erectile dysfunction is uncommon after different techniques of bulbar urethroplasty and is not affected by urethral transection which could be not taken in considering the proper technique decision as transecting and non-transecting techniques have the same success rate.

## Data Availability

The datasets used during this study are available from the corresponding author on reasonable request.
